# Activation of the ERK1/2 Signaling Pathway during the Osteogenic Differentiation of Mesenchymal Stem Cells Cultured on Substrates Modified with Various Chemical Groups

**DOI:** 10.1155/2013/361906

**Published:** 2013-08-28

**Authors:** Bing Bai, Jin He, Yan-Shu Li, Xiu-Mei Wang, Hong-Jun Ai, Fu-Zhai Cui

**Affiliations:** ^1^Department of Prosthodontics, School of Stomatology, China Medical University, Shenyang 110001, China; ^2^State Key Laboratory of New Ceramics and Fine Processing, Department of Materials Science and Engineering, Tsinghua University, Beijing 100084, China; ^3^Department of Cell Biology, Key Laboratory of Cell Biology, Ministry of Public Health and Key Laboratory of Medical Cell Biology, Ministry of Education, China Medical University, Shenyang 110001, China

## Abstract

The current study examined the influence of culture substrates modified with the functional groups –OH, –COOH, –NH_2_, and –CH_3_ using SAMs technology, in conjunction with TAAB control, on the osteogenic differentiation of rabbit BMSCs. The CCK-8 assay revealed that BMSCs exhibited substrate-dependent cell viability. The cells plated on –NH_2_- and –OH-modified substrates were well spread and homogeneous, but those on the –COOH- and –CH_3_-modified substrates showed more rounded phenotype. The mRNA expression of BMSCs revealed that –NH_2_-modified substrate promoted the mRNA expression and osteogenic differentiation of the BMSCs. The contribution of ERK1/2 signaling pathway to the osteogenic differentiation of BMSCs cultured on the –NH_2_-modified substrate was investigated *in vitro*. The –NH_2_-modified substrate promoted the expression of integrins; the activation of FAK and ERK1/2. Inhibition of ERK1/2 activation by PD98059, a specific inhibitor of the ERK signaling pathway, blocked ERK1/2 activation in a dose-dependent manner, as revealed for expression of Cbf**α**-1 and ALP. Blockade of ERK1/2 phosphorylation in BMSCs by PD98059 suppressed osteogenic differentiation on chemical surfaces. These findings indicate a potential role for ERK in the osteogenic differentiation of BMSCs on surfaces modified by specific chemical functional groups, indicating that the microenvironment affects the differentiation of BMSCs. This observation has important implications for bone tissue engineering.

## 1. Introduction

Bone tissue engineering is an attractive approach with great potential for repairing bone defects. The basic strategy of tissue engineering for the structural and functional restoration of damaged bone involves the use of artificial biomaterials as scaffolds for the culture of specific types of cells, which are then stimulated with growth factors [1]. On the basis of their promising osteogenic differentiation ability, bone marrow stromal cells (BMSCs) have been widely used as seed cells in bone tissue engineering, either *in vitro *or* in vivo* [2, 3]. The hypothesis that stem-cell differentiation can be induced by parameters, including structure, chemistry, mechanic, and molecule delivery of biomaterials, has already been substantiated [4–11]. Biomaterial technologies provide the exciting possibility of deconstructing and then reconstructing niches, allowing quantitative analysis of stem-cell behavior in a manner not previously possible [4].

Current research has confirmed that the surface chemistry of the substrates influences the adsorption and the conformation of extracellular matrix protein, such as fibronectin (FN) [12]. Binding of specific integrin adhesion receptors to this protein modulates the activity of focal adhesion kinase (FAK) and the intracellular signaling cascades of osteoblast- and myoblast-like cells [13, 14]. As one of the primary transducers of integrin signals to the cell nucleus, the mitogen-activated protein kinase (MAPK) pathway provides a plausible link between cell surface integrin activation and subsequent stimulation of core binding factor alpha 1 (Cbf*α*-1) in osteoblast-like cells [15, 16]. Extracellular signal-related kinase (ERK1/2) is an important member of the MAPK family of serine/threonine protein kinases [17]. Appropriate soluble and insoluble signals can activate ERK1/2, which then activates important cellular processes, including key transcriptional and phenotypic differentiation programs via sequential activation of protein kinase cascades [18]. The activation of the ERK1/2 pathway has been reported to be involved in the osteogenic differentiation of BMSCs and differentiation of the 3T3L1 preadipocyte cell line into mature adipocytes [19–21]. The regulation of ERK1/2 also contributes to the balance between the osteogenesis and adipogenesis of human adipose-derived stem cells (hASCs) [18, 22]. However, the mechanism by which the surface chemistry of the substrate influences osteogenesis of BMSCs is still unknown, and the effects of ERK1/2 pathway on the stem-cell fate are also unclear. To investigate these processes, we used self-assembled monolayers (SAMs) of alkanethiolates on gold surfaces to evaluate the effects of surface chemistry on the stem cells. Previous studies of biomaterials have routinely used protein-coated dishes to mimic the cellular microenvironment and to elucidate the functions of the matrix in regulating cellular processes. However, the adsorption of proteins is a complex process and often proceeds with a lack of control over the orientation and conformation of proteins at the surface. It is therefore difficult to control the biological activities of proteins adsorbed to man-made materials, and it is impractical to use these substrates as models of the extracellular matrix (ECM) [23]. SAMs, a recently developed technology, offer a set of monolayers which surface chemistry characteristics are well suited to the studies of cell-ECM interactions. Using SAMs technology, previous studies have demonstrated that surface chemistry regulates the specific differentiation of stem cells [24–28].

In the current study, we used a novel SAMs technology to precisely control the density of specific functional groups on the substrate. Multiple phenotypic features of rabbit BMSCs, including initial adhesion, morphology, long-term growth, gene expression, and functional differentiation, were examined. We also analyzed the relationship between the osteogenic differentiation of BMSCs and activation of the ERK1/2 pathway as a function of culture on SAMs substrates presenting different surface chemistries. These results provide support for future studies to identify surface chemistry parameters to achieve the optimal control over stem-cell osteogenic differentiation. 

## 2. Materials and Methods

### 2.1. Preparation and Characterization of SAMs of Alkanethiolates on Gold

Glass coverslips (15 mm diameter) were washed with ethanol in an ultrasound bath, followed by rising with double-distilled water. Gold coated substrates were prepared by sequential deposition titanium (10 nm) and gold (40 nm) film via an electron beam deposition (ANELVA L-400EK, Japan). The gold-deposited films were immersed in ethanol solution containing 1 mM 11-mercapto-1-undecanol (–OH, Sigma, USA), 11-mercaptoundecanoic acid (–COOH, Sigma, USA), 11-amino-1-undecanethiol (–NH_2_, Sigma, USA) and 1-Undecanethiol (–CH_3_, Sigma, USA). Following overnight assembly, the substrates were thoroughly rinsed with ethanol and distilled water. Finally, these substrates were placed in 24-well tissue culture polystyrene plates (Corning, USA), sterilized with 75% alcohol overnight, and then rinsed extensively with sterilized PBS [29].

Ambient air-water-substrate contact angle measurements (4 *μ*L of ultrapure water) were taken with the OCA20 contact angle system (Dataphysics, German) fitted with a digital camera and analyzed using in-house image analysis software. Photoelectron Spectroscopy (XPS) analysis was carried out on Escalab 250 Xi (VG, UK) with Carbon (284.8 eV) as a marker, and the examined area was 300 *μ*m × 700 *μ*m. The surfaces with different terminal functional groups were observed using an atomic force microscope (MFP-3D-S, Asylum Research, USA) in contact mode under an air atmosphere. The photographs were taken under the open-loop condition, and an Olympus AC240TS probe with Si3N4 cantilevers was used.

### 2.2. Culture and Harvest of BMSCs

Rabbit bone marrow stromal cells (BMSCs) were isolated from the tibias of 4-month-old New Zealand white rabbits. Briefly, the tibia of an anesthetized rabbit was punctured with a 16-gauge needle, and 4-5 mL of bone marrow was aspirated into a 10 mL syringe containing 5000 units of heparin. The bone marrow was filtered through a cell strainer (40 *μ*m), and the cells were resuspended and cultured in Dulbecco's modified Eagle's medium (DMEM, Gibco, Rockville, MD, USA) containing 10% fetal bovine serum (FBS, Hyclone, Logan, UT, USA), 100 IU/mL penicillin G, and 100 *μ*g/mL streptomycin. The medium was changed every 2 days. When 70–80% of the bottom of the dish was covered by the cell monolayer, the cells were enzymatically harvested and centrifuged at 800 g for 5 min. All experiments were conducted using cells below passage 4. The cells were then reseeded in standard growth medium at 5 × 10^4^ cells/cm^2^ onto substrates modified with different chemical functional groups and cultured to permit attachment and spreading.

### 2.3. Cell Viability Assay

Cell viability was determined with the CCK-8 cell viability assay (Dojindo Laboratories, Kumamoto, Japan) according to the manufacturer's protocol. Briefly, after incubation at 37°C/5% CO_2_ for 1, 3, 5, and 7 days, the culture medium was replaced with 300 *μ*L of medium containing 30 *μ*L of CCK-8 per well, and the cells were incubated for 3 h in the incubator. Finally, the cell viability was measured as the absorbance at 450 nm using a microplate reader (Bio-Rad, Model 680, Hercules, CA, USA).

### 2.4. Immunofluorescence Assay

The cells were seeded in standard growth medium (1 × 10^4^ cells/cm^2^) onto surfaces modified with the various chemical functional groups. After incubation for 1 and 3 days, the cells were rinsed 3 times with ice-cold PBS, fixed with 4% paraformaldehyde for 15 min, permeabilized with 0.1% Triton X-100 for 10 min, and then blocked with normal goat serum for 30 min to prevent nonspecific binding. The cells were stained with FITC phalloidin (1 : 100, Sigma, St. Louis, MO, USA) and antivinculin (1 : 100, Sigma) for 2 h, followed by Alexa Fluor 594 conjugated goat anti-mouse IgG (H + L) (Invitrogen, Carlsbad, CA, USA) and counterstained with 4,6-diamidino-2-phenylindole (DAPI, Sigma). These cells were visualized by indirect fluorescence using a laser confocal scanning microscope (Olympus FV1000S- SIM/IX81). 

### 2.5. Real-Time RT-PCR

After 1, 3, 5, 7, 10, and 14 days of culture, the cells were harvested, and total RNA was extracted using the Trizol reagent (Invitrogen). The RNA concentrations were determined spectrophoto-metrically at 260 nm prior to reverse transcription. Real-time RT-PCR was performed on a quantitative real-time amplification system (MxPro-Mx3000, Stratagene, La Jolla, CA) using a SYBR PrimeScript RT-PCR Two-Step Kit (TaKaRa, Otsu, Shiga, Japan) according to the manufacturer's instructions. Equal amounts of total RNA (1 *μ*g) from each sample were converted into cDNA in a reverse transcription reaction. PCR for each gene was carried out in a 20 *μ*L reaction mixture containing 2 *μ*L cDNA template. The PCR conditions were 95°C for 1 min followed by 40 cycles of 95°C for 10 s and 60°C for 40 s. The 2^−ΔΔCT^ method was used to calculate the relative levels of gene expression using Strata gene MxPro QPCR software v3.00 (Stratagene). The housekeeping gene GAPDH expression was used as the internal control. A standard melting-curve cycle was used to confirm the quality of amplification. The reactions were performed in triplicate for each sample. The gene-specific primers were designed with the Primer premier 5 software (Premier Biosoft International, Palo Alto, CA, USA), as presented in Table 1.

### 2.6. Western Blot Analysis

At predetermined time points, the cells were washed 3 times with ice-cold PBS and lysed on ice for 20 min. Lysis buffer contained 50 mM Tris-HCl, 150 mM NaCl, 0.2 g/L sodium azide, 0.1 g/L phenylmethylsulfonyl fluoride, 1 g/L sodium dodecyl sulfate (SDS), 10 g/L NP-40, 5 g/L sodium-deoxycholate, and 0.1 g/L aprotinin (pH 8.0). The lysates were centrifuged (12,000 rpm for 10 min at 4°C), and protein quantitation was carried out using the BCA protein assay (R&D Systems, Minneapolis, MN, USA). A sample of 40 *μ*g of protein from each group was resolved by 10% SDS-polyacrylamide gel electrophoresis and transferred onto polyvinylidene fluoride (PVDF) membranes (Amersham LifeSciences, Arlington Heights, IL, USA) by electroblotting. The membranes were probed with specific primary antibodies followed by horseradish peroxidase-conjugated secondary antibodies and analyzed. The primary antibodies were polyclonal rabbit anti-integrin *α*5 (H-104) (sc-10729, 1 : 1000, Santa Cruz Biotechnology, USA), polyclonal rabbit anti-integrin *αV* (H-75) (sc-10719, 1 : 1000, Santa), polyclonal rabbit anti-integrin *β*1 (M-106) (sc-8978, 1 : 1000, Santa), polyclonal rabbit anti-FAK (no. 3285, 1 : 1000, Cell Signaling Technology, USA), monoclonal rabbit anti-p-FAK (Tyr397) (no. 8556, 1 : 1000, Cell Signaling Technology), monoclonal rabbit anti-ERK1/2 (no. 4695, 1 : 1000, Cell Signaling Technology), monoclonal rabbit anti-p-ERK1/2 (Thr202/204) (no. 4377, 1 : 1000, Cell Signaling Technology), and GAPDH antibodies (sc-25778, Santa, USA). The primary antibodies were detected using horseradish peroxidase-conjugated goat anti-rabbit IgG (1 : 5000, Zhongshan Biotechnology Co., Beijing, China) in blocking solution. The immunoreactive protein bands were detected with an enhanced chemiluminescence (ECL) kit (Pierce ECL, ThermoFisher Scientific, Boston, Massachusetts, USA) and quantified by densitometry according to the manufacturer's instructions.

### 2.7. Statistical Analysis

All experiments were performed in triplicate, and representative results are presented as the means ± standard deviations. The statistical analyses for quantitative assays were performed using the SPSS 11.0 software (SPSS, Inc., Chicago, IL, USA). *P* < 0.05 was considered statistically significant. 

## 3. Results

### 3.1. Physicochemical Characterization of the Different Chemical Functional Groups

The water contact angle measurements of the four alkanethiol-terminated SAMs with functional groups (HS–(CH_2_)_11_X, X = –COOH, –NH_2_, –OH, and –CH_3_) were utilized, and the values of water contact angles were determined as previously described: –OH <–COOH <–NH_2_ <–CH_3_ [29–32]. 

The ratio of S/Au atoms analyzed by XPS and the XPS spectra demonstrated that the four types of functional groups successfully self-assembled. The four surfaces had similar S/Au ratios with values of approximately 0.22, indicating the similar surface density of functional groups. AFM images displayed the same morphology of these functional groups surfaces. The distance between neighboring functional group was approximately 0.5 nm, corresponding with the results of Widrig et al. [33]. The AFM images indicated that the four functional groups consisted of the well-known $\left(\sqrt{3}\times \sqrt{3}\right)$ R30° structure unit, and the densities of four functional groups were 5 × 10^18^/m^2^ [31].

### 3.2. Cell Viability on the Different Chemical Functional Groups

**Figure 1:**
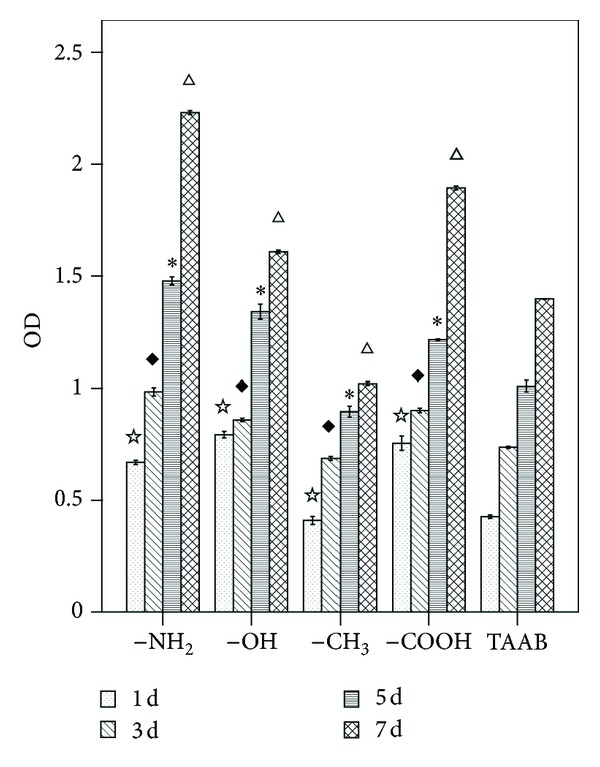
CCK-8 analysis of BMSCs cultured on different chemical functional groups after 1, 3, 5, and 7 days. ^☆^ *P* < 0.05 versus TAAB at day 1; ^*◆*^ *P* < 0.05 versus TAAB at day 3; *P < 0.05 versus TAAB at day 5; ^△^ *P* < 0.05 versus TAAB at day 7.

The effects of modifying the substrates with the different chemical functional groups on the proliferative activity of BMSCs were assessed by CCK-8 analysis.  Figure 1 showed the CCK-8 conversion by BMSCs cultured on the various types of SAMs on days 1, 3, 5, and 7, in conjunction with a clean glass (TAAB) control. On day 1, the levels of cell viability on the –NH_2_, –OH, and –COOH substrates were significantly higher than that on TAAB; the levels of cell viability on the –CH_3_ substrates were significantly lower than that on TAAB. On days 3, 5, and 7, the cell viability significantly upregulated compared to their respective values on day 1 for cells grown on all of the chemically modified substrates. The greatest increase in cell viability was observed on the –NH_2_ substrate, for which the value on day 7 was approximately 4-fold higher than the value observed on day 1. In contrast, the smallest upregulation in cell viability was observed on the –CH_3_ substrate, for which the value after 7 days of cultures was approximately twice that on day 1. The BMSCs exhibited substrate-dependent cell viability of –NH_2_ > –COOH > –OH > TAAB > –CH_3_.

### 3.3. Cytoskeleton and Focal Adhesions on the Different Chemical Functional Groups

**Figure 2:**
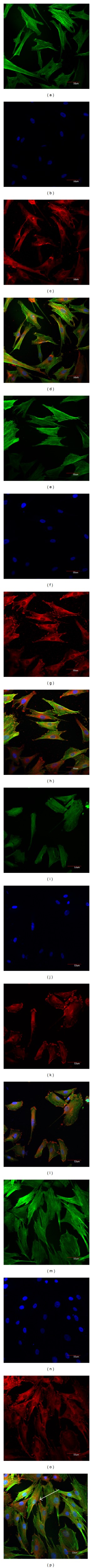
Confocal fluorescence microscopy of the cytoskeleton demonstrating the differentiated cell phenotypes from BMSCs cultured on the various test surfaces after 1 and 3 d of culture. Immunofluorescence staining of anti-F-actin (Green: (a), (e), (i), (m)), DAPI nuclear staining (Blue: (b), (f), (j), (n)), antivinculin (Red: (c), (g), (k), (o)) and merged images ((d), (h), (l), (p)). Scale bar = 50 *μ*m. –NH_2_-modified substrate for 1 day ((a), (b), (c), (d)) and 3 days ((e), (f), (g), (h)), –OH-modified substrate for 1 day ((i), (j), (k), (l)) and 3 days ((m), (n), (o), (p)).

**Figure 3:**
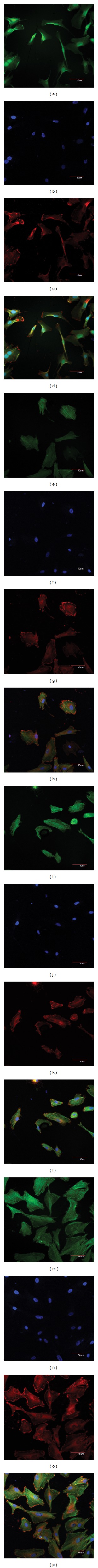
Confocal fluorescence microscopy of the cytoskeleton demonstrating the differentiated cell phenotypes from BMSCs cultured on the various test surfaces after 1 and 3 d of culture. Immunofluorescence staining of anti-F-actin (Green: (a), (e), (i), (m)), DAPI nuclear staining (Blue: (b), (f), (j), (n)), antivinculin (Red: (c), (g), (k), (o)), and merged images ((d), (h), (l), (p)). Scale bar = 50 *μ*m. –CH_3_-modified substrate for 1 day ((a), (b), (c), (d)) and 3 days ((e), (f), (g), (h)), –COOH-modified substrate for 1 day ((i), (j), (k), (l)) and 3 days ((m), (n), (o), (p)).

Figures 2 and 3 showed the typical morphologies of BMSCs on the different chemical substrates. The cytoskeletal structure was examined using a green-fluorescent F-actin stain, and FAK structure was examined using anti-vinculin antibody stained using a red fluorophore. The cells plated on –NH_2_- and –OH-modified substrates were well spread and homogeneous with the actin organized into stress fibers and demonstrated long microtubules and distinct focal adhesions. In contrast, the cells on the –COOH- and –CH_3_-modified substrates showed a more rounded phenotype.

After 1 day, the cells cultured on the –NH_2_- and –OH-modified substrates demonstrated good surface coverage and clear evidence of stress fiber and focal contact formation. The images clearly showed that the focal contacts were located at the ends of the stress fibers (Figures 2(h) and 2(p)). The cells cultured in contact with the –NH_2_-modified substrates demonstrated a well-defined cytoskeleton at both day 1 and day 3 (Figures 2(a) and 2(e)), comparable to the cells cultured on the –CH_3_-modified substrate (Figures 3(a) and 3(e)). The morphology of the BMSCs cultured on the –OH-modified surface was similar to that observed on the –NH_2_-modified substrate during the test period (Figures 2(l) and 2(p)).

Cells cultured in contact with the –CH_3_-modified surface demonstrated evidence of a spindled morphology at day 1 (Figures 3(a) and 3(d)), whereas there were increases in cell coverage, stress fiber, and focal contact formation by day 3 on this surface (Figures 3(e) and 3(h)). The morphology of the cells on the –CH_3_-modified surface was less well spread than these on –NH_2_- and –OH-modified surfaces (Figure 3(h)). 

The individual cells cultured in contact with the –COOH-substrate displayed a smaller spread area at day 1 (Figures 3(i) and 3(l)) than those on –NH_2_- and –OH-modified surfaces, with strong evidence of focal contact formation at both days 1 and 3 (Figures 3(l) and 3(p)). 

### 3.4. Chemical Functional Groups Affected the Osteogenic Differentiation of BMSCs

**Figure 4:**
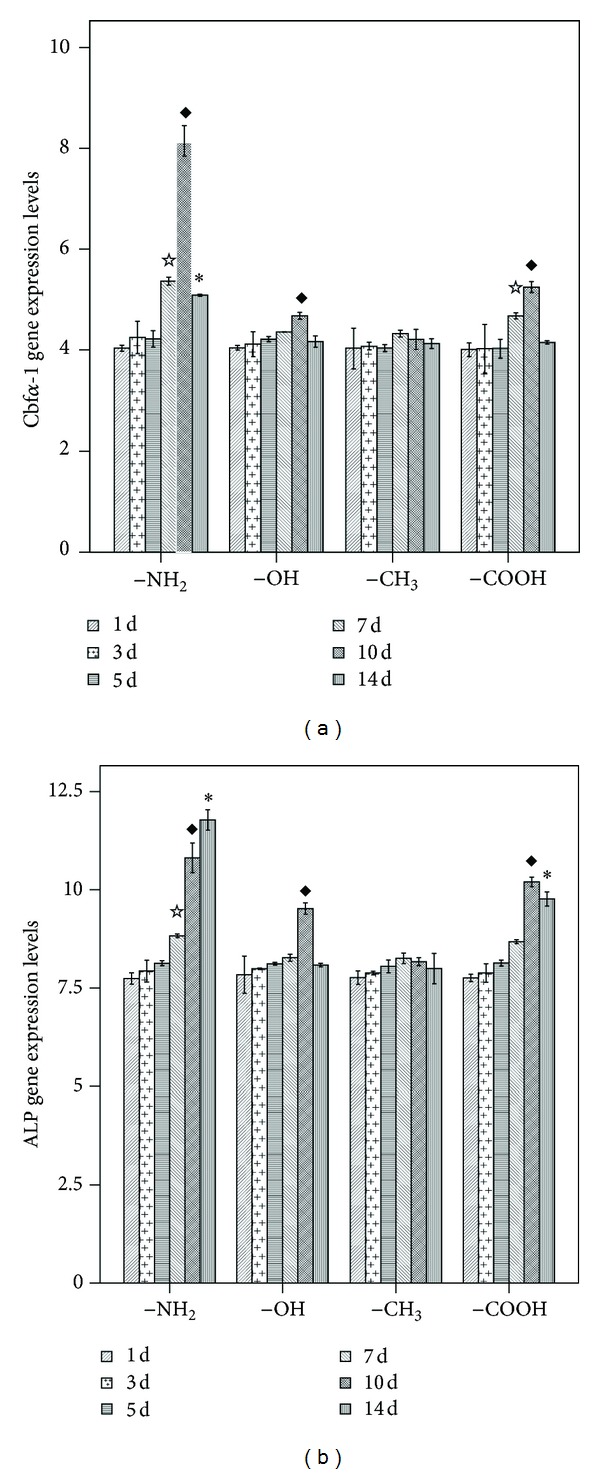
Real-time PCR analysis of the expression of osteogenesis-related genes in BMSCs cultured on surfaces modified with different chemical functional groups. (a) CBFA-1; (b) ALP. ^☆^ *P* < 0.05 versus the respective –CH_3_-modified substrate at 7 d; ^*◆*^ *P* < 0.05 versus the respective –CH_3_-modified substrate at 10 d; *P < 0.05 versus the respective –CH3-modified substrate at 14 d.

The osteogenic differentiation of BMSCs is manifested by numerous specific markers. The most commonly used markers are the synthesis of calcium-binding glycoproteins, osteocalcin and osteopontin; collagen I; the activity of ALP; and the bone tissue mineralization [34]. Because Cbf*α*-1, a potent transcription factor involved in the regulation of several osteoinductive genes, is essential for the differentiation of osteoblasts from mesenchymal precursors and for bone formation [35, 36], we used real-time RT-PCR to examine the expression of Cbf*α*-1 and ALP in BMSCs cultured on the different chemical substrates (Figure 4). All results were normalized to the appropriate GAPDH values of the test samples and are represented as an increase/decrease in cellular expression. 

Significant upregulation of both Cbf*α*-1 and ALP was observed for the cells cultured on the –NH_2_-modified substrate relative to those cultured on the –CH_3_-modified surface. Increased Cbf*α*-1 expression was observed on –NH_2_-modified surface on days 7, 10, and 14. In addition to the effect of the –NH_2_-modified surface, the –OH- and –COOH-modified surfaces caused increases in the expression levels of Cbf*α*-1 and ALP on certain days. For example, increases in both markers were observed in the cells cultured on the –OH-modified surface on day 10 and on the –COOH-modified surfaces on days 10 and 14. However, these trends were not as obvious as those observed for the –NH_2_-modified surface (Figure 4(a)). The expression of ALP was increased for cells cultured on the –NH_2_-modified surface on days 7, 10, and 14. The ALP expression was also increased for the cells cultured on the –OH-modified surface on day 10 and on the –COOH-modified surface on days 10 and 14 (Figure 4(b)).

### 3.5. Activation of the ERK1/2 Pathway in BMSCs Cultured on –NH_2_ and –CH_3_ Substrates

These results confirmed that chemical modifications of the substrate can affect the adhesion, spreading, and osteogenic differentiation of BMSCs. The –NH_2_-modified surfaces promoted osteogenesis of the BMSCs in the absence of biological stimuli as previous described [24–26]. To investigate the downstream signaling pathways in BMSCs that may be involved in the regulation of osteogenesis during culture on the –NH_2_- and –CH_3_-modified substrates (the modifications with the strongest and weakest ability to promote the osteogenic differentiation of BMSCs, resp.), we focused on the key proteins of the ERK1/2 signaling pathway, as suggested by earlier studies implicating these pathways [37]. Cells were cultured on the –NH_2_- and –CH_3_-modified surfaces for up to 14 days. The analysis of the mRNA and lysates of cells revealed that chemical groups influenced the ERK1/2 pathway.

**Figure 5:**
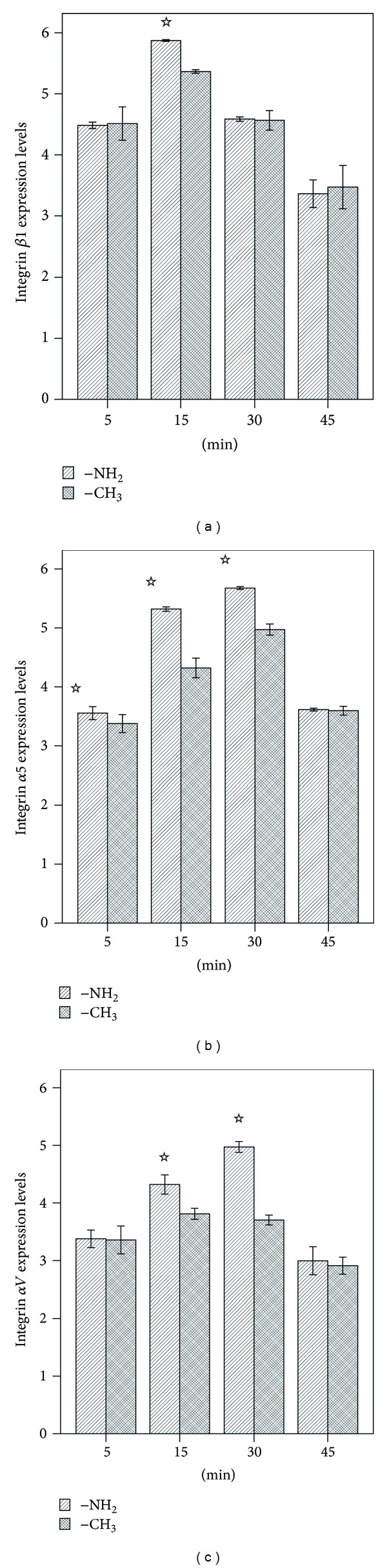
Real-time PCR analysis of the expression of osteogenesis-related genes in BMSCs cultured on the –NH_2_- and –CH_3_-modified substrates. (a) Integrin *β*1, (b) integrin *α*5, (c) integrin *αV*. ^☆^ *P* < 0.05 versus the respective –CH_3_-modified substrate.

Integrins are a family of transmembrane proteins that bind the cell to extracellular matrix proteins, attach to the cytoskeleton at focal adhesion sites, and induce intracellular signals [38–41]. The results of the evaluation of the mRNA expression of integrins *β*1, *α*5, and *αV* in cells cultured on –NH_2_- and –CH_3_-modified substrates are shown in Figure 5. The expression level of integrin *β*1 in cells cultured on the –NH_2_-modified surface was significantly greater than that for cells cultured on the –CH_3_-modified surface at 15 min (Figure 5(a)); integrin *α*5 on the –NH_2_-modified surface was higher than on the –CH_3_-modified surface at 5 min and significantly higher at 15 min and 30 min (Figure 5(b)); the expression of integrin *αV* was significantly higher on the –NH_2_-modified surface than on the –CH_3_-modified surface at 15 min and 30 min (Figure 5(c)). The peak of the expression of integrin *β*1 occurred at 15 min (Figure 5(a)), while the peaks of integrin *α*5 and integrin *αV* expression occurred at 30 min (Figure 5(b)).

**Figure 6:**
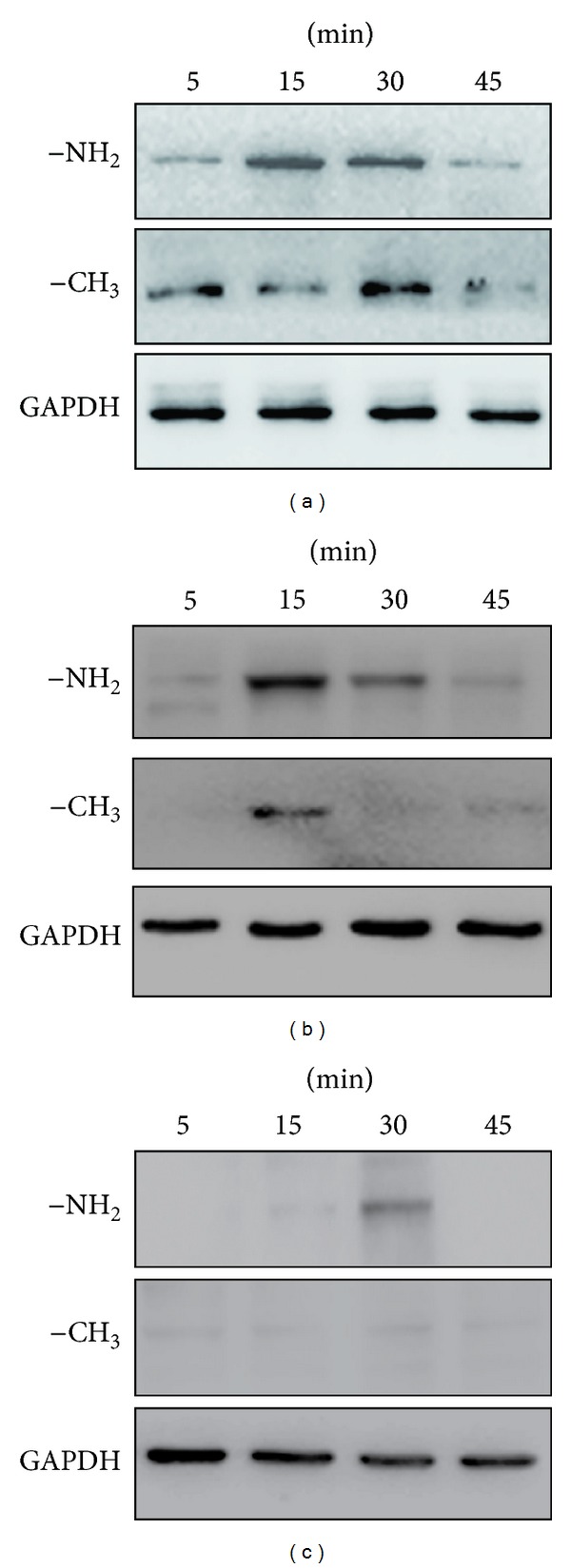
The –NH_2_-modified substrate induces the activation of osteogenesis-related genes. (a) Integrin *β*1, (b) integrin *α*5 and (c) integrin *αV*  in BMSCs. Cells were cultured on the –NH_2_- and –CH_3_-modified substrates, and lysates were prepared at the indicated times. Lysates were subjected to immunoblot analysis using integrin antibodies. An aliquot of each lysate was subjected to a kinase detection assay using SDS-PAGE. GAPDH was used as a control.

Next, we examined integrin activities at 5, 15, 30, and 45 min. As shown in Figure 6, the peak level of integrin *β*1 activity in cells cultured on substrates modified with the –NH_2_ group was observed at 15 min and was followed by a decline in the levels between 15 and 45 min; the peak level of integrin *β*1 activity in cells cultured on substrates modified with the –CH_3_ group was observed at 30 min (Figure 6(a)); the activity of integrin *α*5 on the –NH_2_-modified surface was significantly higher than on the –CH_3_-modified surface at 15 min and 30 min (Figure 6(b)); the activity of integrin *αV* was significantly higher on the –NH_2_-modified surface than on the –CH_3_-modified surface at 30 min (Figure 6(c)).

**Figure 7:**
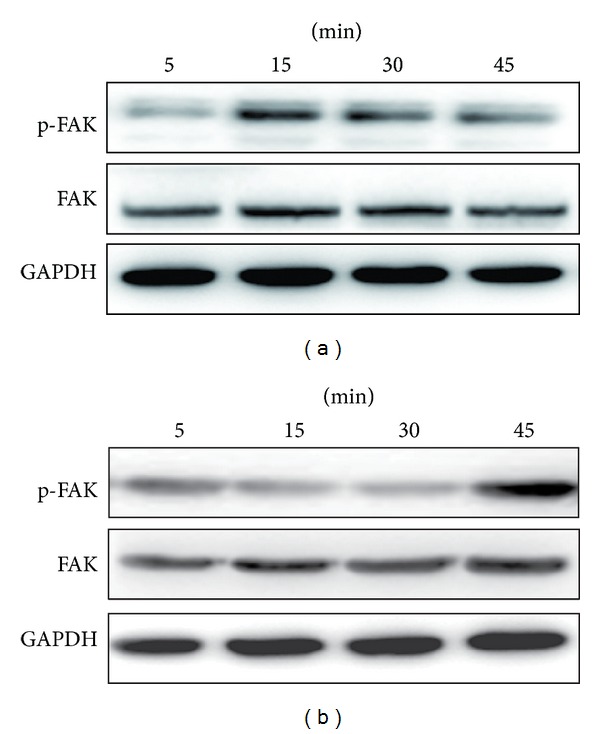
The –NH_2_-modified substrate induces the activation of FAK in BMSCs. Cells were cultured on the –NH_2_-modified substrate (a) and the –CH_3_-modified substrate (b), and lysates were prepared at the indicated times. Lysates were subjected to immunoblot analysis using phosphospecific and nonactivated FAK antibodies. An aliquot of each lysate was subjected to a kinase detection assay using SDS-PAGE. GAPDH was used as a control.

The *α* and *β* subunits of the integrins do not have any intrinsic enzymatic activity. However, these proteins interact with cytoplasmic proteins that nucleate the formation of large protein complexes. These complexes in turn activate intracellular signaling cascades, leading to alterations in gene expression. Focal adhesion kinase (FAK) is one of the numerous signaling molecules present in focal adhesion sites [42]. We examined FAK activity at 5, 15, 30, and 45 min. As shown in Figure 7, the peak level of FAK activity in cells cultured on substrates modified with the –NH_2_ group was observed at 15 min and was followed by a decline in the levels between 15 and 45 min. The peak level of FAK activity in cells cultured on substrates modified with the –CH_3_ group was observed at 45 min, but almost no detectable activation of FAK could be measured at 5 and 15 mins. Taken together, these results indicated that the –NH_2_-modified substrate promoted the activation of FAK.

**Figure 8:**
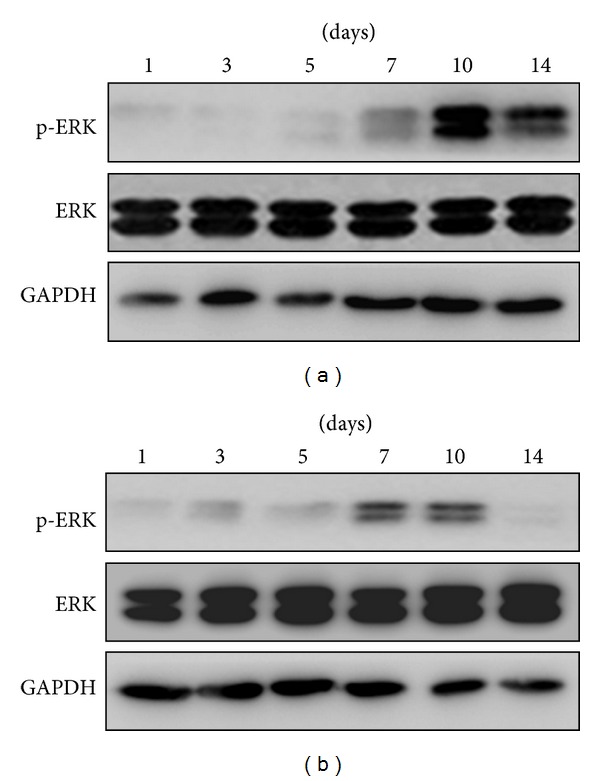
The –NH_2_-modified substrate induces the activation of ERK1/2 in BMSCs. Cells were cultured on the –NH_2_-modified substrate (a) and the –CH_3_-modified substrate (b), and lysates were prepared at the indicated times. Lysates were subjected to immunoblot analysis using phosphospecific and nonactivated ERK1/2 kinase antibodies. An aliquot of each lysate was subjected to a kinase detection assay using SDS-PAGE. GAPDH was used as a control.

To investigate whether the osteogenic differentiation of BMSCs on the –NH_2_-modified surface paralleled the activation of ERK1/2, we examined ERK1/2 activity on days 1, 3, 5, 7, 10, and 14. As shown in Figure 8, the chemically modified substrates did not affect ERK1/2 phosphorylation until 7 days. A high level of ERK activity was observed on day 10, which was followed by a decline. The time period during which phosphorylation of ERK1/2 was the highest was associated with high expression of Cbf*α*-1 and ALP. Meanwhile, the activation of ERK on the –CH_3_-modified substrate at each time point was less than on the –NH_2_-modified substrate. Taken together, these results indicated that the osteogenic differentiation of BMSCs on the –NH_2_-modified surface correlated well with the activation of ERK1/2. 

**Figure 9:**
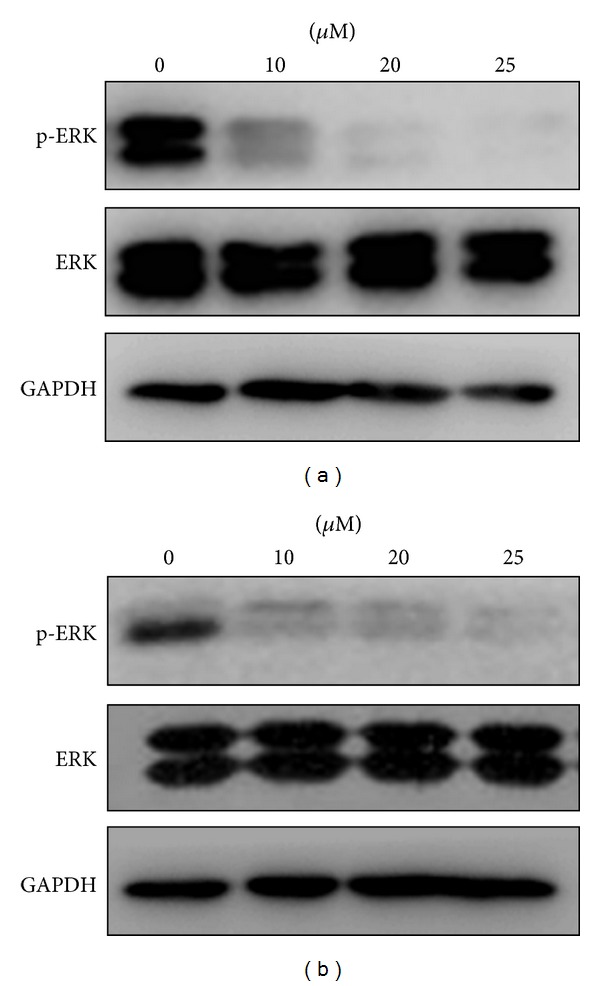
PD98059 inhibits ERK1/2 activation in a dose-dependent manner. Cells were cultured on the –NH_2_- and –CH_3_-modified substrates containing 0, 10, 25, and 50 *μ*M of PD98059 for 10 days. (a) –NH_2_-modified substrate and (b) –CH_3_-modified substrate. Cell lysates were analyzed by immunoblots using phospho-ERK-specific and nonactivated ERK1/2 antibodies; GAPDH was used as a control.

To clarify whether ERK activation is necessary for osteogenic differentiation of the BMSCs cultured on the –NH_2_-modified substrate, we blocked the ERK1/2 signaling pathway using different amounts of PD98059, a potent and selective inhibitor of MEK1 (a kinase upstream of ERK1/2) [43]. To determine whether PD98059 suppressed the increases in ERK1/2 activity observed during culture of the cells on the –NH_2_- and –CH_3_-modified substrates, we measured the levels of ERK1/2 and p-ERK1/2 in the presence of PD98059 at concentrations of 0, 10, 25, and 50 *μ*M on day 10. As shown in Figures 9(a) and 9(b), treatment of BMSCs with PD98059 (0, 10, 25, and 50 *μ*M) resulted in concentration-dependent inhibition of ERK activation on day 10. 

**Figure 10:**
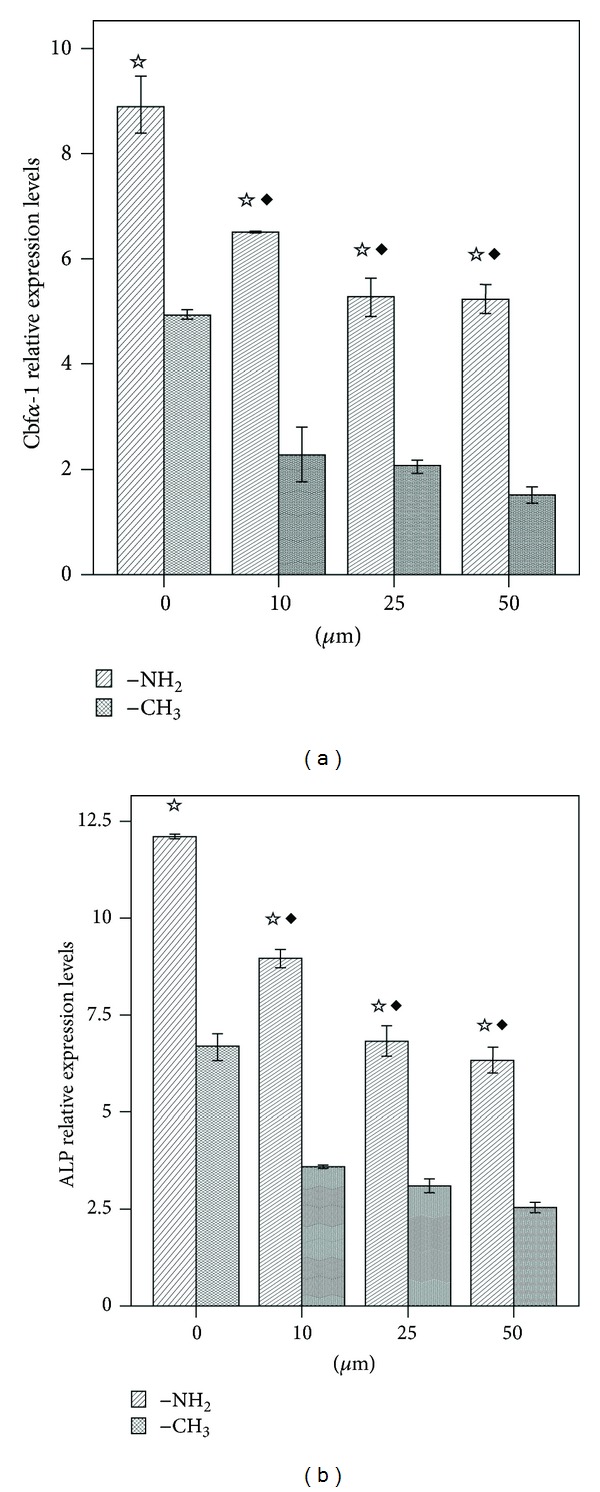
Real-time RT-PCR analysis of the expression of osteogenesis-related genes in BMSCs cultured on the –NH_2_- and –CH_3_-modified substrates in the presence of 0, 10, 25, and 50 *μ*M of PD98059 for 10 days. (a) Cbf*α*-1 and (b) ALP. ^☆^ *P* < 0.05 versus the respective –CH_3_-modified substrate; ^*◆*^ *P* < 0.05 versus the 0 *μ*M dose group.

Osteogenic differentiation of BMSCs was assessed on the basis of Cbf*α*-1 and ALP expression using real-time RT-PCR. As shown in Figures 10(a) and 10(b), treatment of BMSCs with PD98059 at day 10 resulted in concentration-dependent inhibition of Cbf*α*-1 and ALP activities. However, on day 10, the Cbf*α*-1 and ALP activities in each set of samples cultured on the –NH_2_-modified substrate in the presence of the same concentrations of PD98059 were still significantly higher than that in the corresponding groups cultured on the –CH_3_-modified substrate (*P* < 0.05). On the basis of the western blot analysis, the inhibitory effect of PD98059 on the phosphorylation of ERK1/2 paralleled the downregulation of Cbf*α*-1 and ALP activities in a similar dose-dependent manner (Figures 9 and 10).

## 4. Discussion

Cell-material interactions are critical for many biomedical and biotechnological applications. What makes these cells different when they are adhered to different materials? To answer that question, we must first fully understand how the cells translate the cues they receive from the extracellular microenvironment to the intracellular signaling, which eventually leads to activation of transcription factors and altered gene expression [44]. 

A series of reviews recently summarized numerous studies that clearly show that the biochemical characteristics of the substrate, as well as its rigidity and spatial organization, are recognized by cells through differential signaling from integrin-based molecular complexes [45–47]. Cells interact with solid substrates such as extracellular matrix (ECM) proteins through integrins, which, upon phosphorylation, initiate signaling cascades, many of which alter the activity of transcription factors that control gene expression. Integrin-ECM binding induces a wide range of intracellular signaling events such as the activation of Ras, FAK, Src, Rac/Rho/CDC42 GTPases, MAPK, PKC, and PI3K in different cell systems [48–50]. The pathway from integrin binding to FAK to MAPK is a particularly well-studied pathway, but there are a large number of pathways by which microenvironmental cues are transmitted into the cell and eventually alter gene expression.

The chemical characteristics of the substrate surface have been shown to affect the biological behavior in different cell systems in general, and the osteogenic differentiation potential of BMSCs *in vitro* in particular, in a number of studies [24–26]. However, the molecular mechanism underlying the substrate-dependent osteogenic commitment of these cells remains unclear. Elucidation of these mechanisms could improve the ability to promote the osteogenic differentiation of BMSCs for the purposes of cell therapy and tissue engineering. As one of the major components of the MAPK family, ERK1/2 has been associated with cellular survival, proliferation, and differentiation and especially the osteoblastic differentiation of BMSCs [51–53]. Thus, the objective of this study was to investigate whether activation of the ERK1/2 pathway was involved in the differentiation of BMSCs cultured on differentially modified surfaces into an osteogenic lineage.

It is important to recognize that the same intracellular signaling molecules that are activated by integrins are also activated by the stimulation of various cytokines and other cues [54]. Cell behavior is often dependent on synergy between growth factor/cytokine-mediated and integrin-mediated signaling pathways [55–57]. The well-defined regulatory effect of dexamethasone (Dex), present in osteogenic medium (OM), on cellular growth and differentiation is usually assumed to contribute to the mitogenesis and osteogenic differentiation of BMSCs. This mediator has previously been reported to activate the ERK1/2 pathway [19]. To eliminate the osteogenic differentiation effect of Dex, we cultured BMSCs in the absence of Dex and evaluated the osteogenesis of BMSCs. The biological behavior of BMSCs has been previously described [58]. 

SAMs are currently the best available class of model organic surfaces with which to control the interfacial structure and properties. Previous studies have demonstrated that terminal chemical groups control cell behavior including adhesion, migration, and differentiation in various cell types including human osteoblast-like cells [59], human fibroblasts [60], mesenchymal stem cells [24–26], and neural stem cells [27]. We have developed molecules with specific functional groups by a novel SAM technology and confirmed the structures produced by these functional groups by AFM and XPS. XPS spectra demonstrated that the four types of functional groups successfully self-assembled, and AFM images displayed the same morphology of these functional group surfaces [30, 31]. Because the different chemical functional groups had same structures and same elements, the only reason of the different behaviors of cells seeded on different chemical functional groups was their chemistry.

It is well established that cell adhesion and morphology influence subsequent activities [61]. In accordance with previous publications, our study confirms that cell viability depends on surface properties and varies with individual functional groups rather than general surface properties such as hydrophilicity [62–64]. For example, the –OH-modified substrate was the most hydrophilic surface (Figure 1), but similar cell contact areas were observed on this substrate and on the –NH_2_-modified surface (Figure 2). Figure 2 showed that the cells plated on –NH_2_- and –OH-modified substrates were well spread and homogeneous with the actin organized into stress fibers and demonstrated long microtubules and distinct focal adhesions. On the other hand, the cell viability results demonstrated that all of the substrates maintained levels of viable cell adhesion throughout the test periods in all of the mediums tested. However, BMSCs cultured on the –NH_2_ substrate maintained increased viable cell adhesion on day 7.

Consistent with the studies of JM Curran, which indicated that the surface properties of the culture substrate influence the phenotype of BMSCs [24, 25], our gene expression analysis using real-time RT-PCR suggests that BMSCs cultured on –NH_2_-modified substrates in the absence of osteoinductive medium upregulate the expression of Cbf*α*-1 and ALP during the first 14 days of culture (Figure 4). The function of Cbf*α*-1 dominates the osteoblast differentiation, which is characterized by regulation of the expression of major osteogenesis-related genes, including COLI, ALP, and OCN. Cbf*α*-1 also maintains the functions of early-stage differentiation of the osteoblasts [21]. As one of the important markers of osteogenesis assessment, ALP was shown to regulate organic or inorganic phosphate metabolism via the hydrolysis of phosphate esters and to function as a plasma membrane transporter for inorganic phosphates [18]. Based on the results of cell viability, cell adhesion, and the mRNA expression, we affirmed that –NH_2_ chemical functional group could contribute to the osteogenic differentiation of BMSCs. So we selected the –NH_2_-modified substrate to examine the role of ERK1/2 signal pathway in osteogenic differentiation of BMSCs as a function of the chemical groups used to modify the substrate.

Integrin-mediated multiprotein adhesion complexes link the extracellular matrix to the actin cytoskeleton. Molecular analyses of these adhesion sites indicate that the integrin adhesome consists of ~160 distinct components. Examination of the molecular interactions that takes place among the different constituents of the adhesome points to an extraordinary connectivity. The biological activities of the adhesome components are diverse and include several actin regulatory elements that affect the organization of the attached cytoskeleton. Many of the adaptor proteins directly or indirectly link actin to integrins and a wide range of signaling molecules, such as kinases, phosphatases, and G proteins and their regulators. It seems likely that the tight association between the structural and signaling elements of the adhesome provides its unique properties as a sensitive environmental sensing system to the adhesion machinery [65]. Therefore, optimizing the osteogenic differentiation potential may not simply involve providing a uniform coating to engage a distinct receptor, but instead the identification of the surface properties that control the presentation of integrin-specific epitopes associated with specific chemical groups may be required [37].

Based on our findings, *β*1, *α*5, and *αV* integrins-FAK appear to mediate the adhesion of BMSCs mediated by specific chemical groups. These associations may perhaps even be required for osteogenic differentiation (Figures 5, 6, and 7). Figures 5 and 6 showed the results of the expression of integrins *β*1, *α*5, and *αV* in cells cultured on –NH_2_- and –CH_3_-modified substrates. The expression level of integrins *β*1, *α*5, and *αV* in cells cultured on the –NH_2_-modified surface was significantly greater than that for cells cultured on the –CH_3_-modified surface at different times; Figure 7 showed the FAK activity in cells which were cultured on substrates modified with the –NH_2_ group and –CH_3_ group. The peak level of FAK activity in cells cultured on substrates modified with the –NH_2_ group was observed at 15 min and was followed by a decline in the levels between 15 and 45 min, but the peak level of FAK activity in cells cultured on substrates modified with the –CH_3_ group was observed at 45 min, but almost no detectable activation of FAK could be measured at 5 and 15 mins. Taken together, these results indicated that the –NH_2_-modified substrate promoted the activation of integrin and FAK. 

Our results show that the activation of ERK1/2 in BMSCs cultured on the –NH_2_-modified substrate was greater than that on the –CH_3_-modified substrate during the period between 7 and 14 days (Figure 8). The chemically modified substrates did not affect ERK1/2 phosphorylation until 7 days. The expression level of the phosphorylation of ERK1/2 in cells cultured on the –NH_2_-modified surface was significantly greater than that on the –CH_3_-modified surface at 10 and 14 days. In this study, we found that ERK1/2 was activated in a time-dependent manner consistent with the expression of Cbf*α*-1 and ALP in BMSCs cultured on the –NH_2_-modified substrate. Hence, it can be concluded that ERK1/2 activation parallels the osteogenic differentiation of BMSCs on the –NH_2_-modified substrate. 

To further investigate the role of the ERK1/2 pathway in BMSC osteogenic differentiation on the –NH_2_-modified substrate, the activation of the ERK1/2 pathway was blocked with PD98059, and the corresponding osteogenic differentiation of BMSCs was examined on day 10. PD98059 is a specific inhibitor of MEK1, a kinase upstream of ERK1/2 and is able to bind to the inactive forms of MEK1 and thereby block the ERK1/2 signaling pathway [66]. PD98059 inhibited expression of the phosphorylation of ERK1/2 in a dose-dependent manner in both substrates. At a concentration 50 *μ*M of PD98059, expression of the phosphorylation of ERK1/2 was almost completely inhibited (Figure 9).  Figure 10 showed that PD98059 inhibited expressions of Cbf*α*-1 and ALP in a dose-dependent manner also. But at the concentration 50 *μ*M of PD98059, the expressions of Cbf*α*-1 and ALP were only partly inhibited (Figure 10). 

Thus, these results demonstrated that blocking the activation of the ERK1/2 pathway leads to the partial inhibition of the osteogenic differentiation of BMSCs on –NH_2_-modified substrates and further suggests that activation of the ERK1/2 pathway is primarily responsible for the osteogenesis of BMSCs on –NH_2_-modified substrates.

## 5. Conclusion

The data presented here suggest that –NH_2_ chemical functional group could contribute to the osteogenic differentiation of BMSCs, and the influences depend on chemical functional group rather than general surface properties such as hydrophilicity.

The BMSCs which cultured on –NH_2_-modified substrates alter their expression of integrins and FAK and activate the downstream ERK1/2 signaling pathway that is required for osteogenic differentiation. It is believed that the balance of molecular events responding to various extracellular stimuli determines the process of cell type-specific differentiation of multipotent mesenchymal stem cells. Therefore, other signaling pathways, such as c-Jun N-terminal kinase, p38 and PI3K, as well as cross-talk between these pathways, need to be further characterized to explore the detailed mechanisms responsible for the osteogenic differentiation of BMSCs cultured on various chemical groups. 

## Figures and Tables

**Table 1:** Sequence of Primers.

Gene	Primers (F: Forward; R: Reverse)	Amplicon size (bp)
Integrin *α*5	F:TGGACTGGCAGAAGCAGAAGG	195 bp
	R:CCAAGGAGAAGTTGAGTGCGATGT	
Integrin *β*1	F:GTGCTGAAGACTACCCCATC	158 bp
	R:CTCCACAAAAGAGCCAAATC	
Integrin *α*V	F:TCAAGATGGAGCAAAGAC	134 bp
	R:CAGGACCACCAAGAAGTA	
Cbf*α*-1	F:GCCACCTCTGACTTCTGC	107 bp
	R:GAAATGCTTGGGAACTGC	
ALP	F:AACCTGGTGGAGGAGGGC	129 bp
	R:CATGTCTGAGGGCTCAAAGAG	
GAPDH	F:CACTTCGGCATTGTGGAG	131 bp
	R:GAGGCAGGGATGATGTTCT	
